# The similarity between N-terminal targeting signals for protein import into different organelles and its evolutionary relevance

**DOI:** 10.3389/fphys.2015.00259

**Published:** 2015-09-24

**Authors:** Markus Kunze, Johannes Berger

**Affiliations:** Department of Pathobiology of the Nervous System, Center for Brain Research, Medical University of ViennaVienna, Austria

**Keywords:** Peroxisomes, PTS2, targeting signals, preprotein, transit peptide, signal peptide, specificity, ambiguous targeting signals

## Abstract

The proper distribution of proteins between the cytosol and various membrane-bound compartments is crucial for the functionality of eukaryotic cells. This requires the cooperation between protein transport machineries that translocate diverse proteins from the cytosol into these compartments and targeting signal(s) encoded within the primary sequence of these proteins that define their cellular destination. The mechanisms exerting protein translocation differ remarkably between the compartments, but the predominant targeting signals for mitochondria, chloroplasts and the ER share the N-terminal position, an α-helical structural element and the removal from the core protein by intraorganellar cleavage. Interestingly, similar properties have been described for the peroxisomal targeting signal type 2 mediating the import of a fraction of soluble peroxisomal proteins, whereas other peroxisomal matrix proteins encode the type 1 targeting signal residing at the extreme C-terminus. The structural similarity of N-terminal targeting signals poses a challenge to the specificity of protein transport, but allows the generation of ambiguous targeting signals that mediate dual targeting of proteins into different compartments. Dual targeting might represent an advantage for adaptation processes that involve a redistribution of proteins, because it circumvents the hierarchy of targeting signals. Thus, the co-existence of two equally functional import pathways into peroxisomes might reflect a balance between evolutionary constant and flexible transport routes.

## Mechanisms of protein translocation across cellular membranes

In eukaryotic cells, an elaborate endomembrane system separates the cytosolic space^1^ from sealed compartments such as mitochondria, peroxisomes, chloroplasts (in plants), and the secretory (endoplasmic reticulum (ER), golgi, trans golgi network (TGN), and lysosome) and endosomal (early and late endosomes) system, which we generally summarize as organelles within this review. The individual compartments of the secretory and endosomal system are interconnected, whereas chloroplasts, mitochondria, and peroxisomes are considered more separate in spite of an exchange of metabolites and membrane constituents between these organelles. This separation can serve various functions such as the local enrichment of specific metabolic intermediates, the sequestration of toxic compounds or the separation of oppositely directed reactions (e.g., biosynthesis and degradation of fatty acids). Some of the enzymatic activities that cohabitate the same organelle cooperate in coupled reactions within certain metabolic pathways to perform complex reactions such as respiration (in mitochondria), photosynthesis (in chloroplasts), or the degradation of fatty acids (in peroxisomes and mitochondria). This implies that the co-localization of diverse enzymes within the same organelle is a prerequisite for an efficient metabolic flux of compounds that are degraded or synthesized. Thus, the proper distribution of proteins among different subcellular compartments is essential for the functionality of a cell. While nearly all proteinaceous components of peroxisomes, mitochondria, or chloroplasts are synthesized by cytosolic ribosomes and transported into the organelles by specific import machineries, the ER is the entrance site for proteins destined for any place along the secretory or endosomal pathway. Only a few cellular proteins are encoded by mitochondrial or chloroplast DNA and are synthesized locally without the need to be imported. Thus, the distribution of proteins is critically dependent on a reliable protein transport system, which requires the cooperation between information specifying the cellular destination of an individual protein and cellular transport machineries, which recognize and process all proteins that harbor such information and need to be transported. The destination of individual proteins is encoded within their primary sequence in the form of short peptides called *targeting signals*, which can be considered postal codes necessary and sufficient to determine the intracellular location. These targeting signals are recognized by *receptor proteins*, which are the frontline of the organellar import machinery and initiate transport of their cargo proteins (Blobel and Dobberstein, 1975). The import mechanisms by which soluble proteins are translocated across the membrane(s) of peroxisomes, mitochondria, chloroplasts, or of the ER are remarkably different. However, the targeting signals for mitochondria, chloroplasts, or the ER appear structurally similar, because they all involve an α-helical domain in proximity to the N-terminus. In contrast, the majority of peroxisomal proteins is equipped with a targeting signal that resides at the extreme C-terminus of the protein. However, a peroxisomal destination can also be encoded by an independent second targeting signal that resides proximal to the N-terminus, but occurs less frequently and has attracted less interest (Schatz and Dobberstein, 1996; Fujiki et al., 2014).

In this review, we compare the different import systems translocating soluble proteins from the cytosol into the lumen of peroxisomes, mitochondria, chloroplasts, or the ER. The receptor proteins of these transport systems all recognize targeting signals encoded within N-terminal sequences that involve an α-helical domain. In particular, we highlight the recent finding that the second peroxisomal targeting signal (PTS) is also encoded by a sequence element that forms an α-helical domain. The similarity to other N-terminal targeting signals distinguishes this PTS (PTS2) from the predominant PTS (PTS1) residing at the C-terminus, which could serve as explanation for the existence of two completely independent PTS that exceeds simple redundancy. In this context, we discuss the specificity of targeting signals, the hierarchy of transport routes and the possibility to change the subcellular location of a protein in evolutionary adaptation processes.

## Mechanisms of protein import from the cytosol into endomembrane systems

Complex protein machineries guide newly generated soluble proteins equipped with suitable targeting signals across the single membrane of peroxisomes and the ER and across the double membrane of chloroplasts and mitochondria. Although these transport machineries act on membrane proteins as well, we restrict ourselves to transport routes of soluble proteins, because this allows a comparison of different organelles within the given space. Moreover, we do not consider further intraorganellar transport processes that act on proteins in the mitochondrial matrix or the chloroplast stroma.

**Figure 1:**
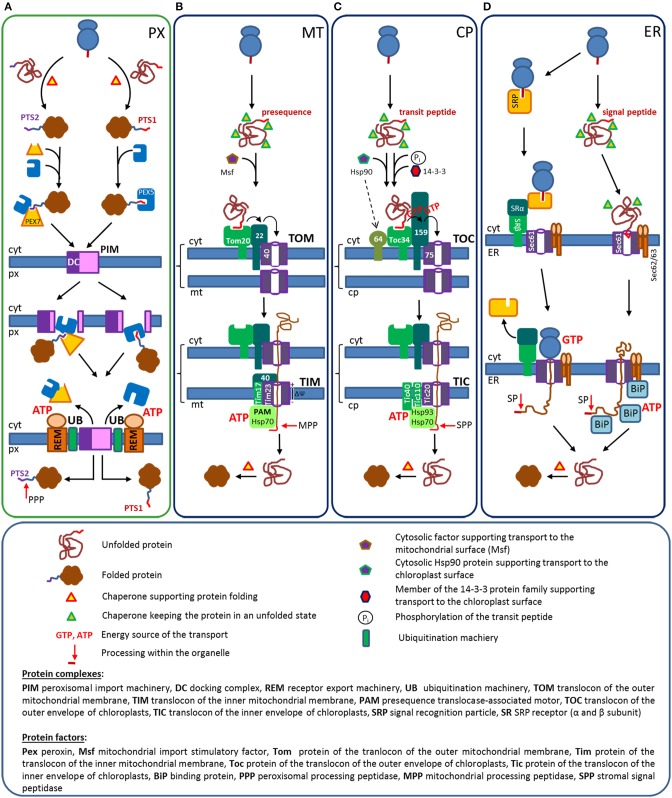
**Protein transport routes from the cytosol into peroxisomes, mitochondria, chloroplasts, and the ER: The transport routes are depicted schematically to highlight certain players^3^**. **(A)** *Peroxisomes*. Proteins encoding either a PTS1 or a PTS2 are folded within the cytosol and interact with the appropriated receptor proteins, Pex5 or the Pex7/co-receptor complex. This induces the translocation of cargo loaded receptors to the docking complex (DC), where they integrate into the peroxisomal membrane and release the cargo into the lumen. Finally, Pex5 and the Pex7/co-receptor complex are ubiquitylated by a specific ubiquitination machinery (UB) and recycled into the cytosol by an ATP driven extraction exerted by the receptor extraction machinery (REM). Soluble proteins reach the peroxisomal matrix in a folded state, but PTS2-carrying proteins are processed by the peroxisomal processing peptidase (PPP). **(B)** *Mitochondria*. Proteins encoding a presequence are translated within the cytosol, but remain in an unfolded state due to their association with proteins of the Hsp70 family. These complexes are transferred by the help of an additional cytosolic factor to a protein complex at the outer mitochondrial membrane (TOM), where the preprotein sequentially interacts with the receptors for soluble proteins (Tom20 and Tom22), before it is handed over to the pore forming translocon (Tom40). The preprotein crosses the outer mitochondrial membrane in an unfolded state and interacts with a protein complex in the inner mitochondrial membrane (TIM). The binding to Tim40 mediates the interaction with the pore forming unit of the inner membrane (Tim23) and the electrochemical gradient (ΔΨ) drags the presequence across the membrane. At the matrix side, the “presequence translocase-associated motor” (PAM)-complex, ropes the preprotein into the matrix by an ATP-driven mechanism that is based on the sequential interaction of mitochondrial chaperones. Next, the N-terminal sequence of the preprotein is cleaved off by the mitochondrial processing peptidase (MPP) and the protein folds within the matrix with the help of mitochondrial folding chaperones. **(C)** *Chloroplasts*. Proteins encoding a transit peptide are translated by cytosolic ribosomes and kept in an unfolded state by proteins of the Hsp70 family. Proteins of the 14-3-3 family, which bind selectively to phosphorylated transit peptides and Hsp90 proteins support the transfer to the chloroplast surface. The outer chloroplast membrane contains multi-protein complexes (TOC) that involve members of two receptor families (Toc34 and Toc159 family), a specific binding factor for Hsp90 proteins (Toc64) and the channel forming translocon Toc75. Transit peptides are translocated via sequential receptor binding from Toc34 to Toc159 and Toc75, which requires the cooperation between the GTPase domains of Toc34 and Toc159. Unfolded preproteins pass the translocon and bind to the multiprotein complex at the inner chloroplast membrane (TIC), involving the pore forming protein Tic20, Tic110, and Tic40, which allow the transfer of the transit peptide across the inner membrane. In the stroma a complex machinery of CpHsp70, Hsp90, and Hsp93, which is attached to the inner side of the chloroplast membrane by the interaction with TIC-proteins, supports the import of the preprotein by an ATPase driven mechanism. Within the chloroplast the transit peptide is cleaved off and the imported proteins are folded. **(D)** *ER*: *Co-translational (left part)*. A functional signal peptide sequence initiates the binding of the signal recognition particle (SRP complex) upon its appearance at the ribosomal exit site. SRP binding stalls translation until the trimeric complex consisting of a nascent chain harboring a signal peptide, a ribosome and a SRP binds to the heterodimeric SRP-receptor (SR) on the surface of the ER. Subsequently, the signal peptide and the ribosome become transferred to the outer side of the Sec61 complex, which is the channel forming translocon. The release of the SRP is coupled to the resumption of translation and the newly synthesized protein is directly inserted into the lumen of the ER. This complex mechanism involves the cooperation of GTPase domains within the SRP and the SR, whereby the hydrolysis of GTP is coupled to diverse conformational changes. However, the major energy consuming step that drives the translocation of preproteins across the ER membrane is the energy of translation (GTP hydrolysis). At the inner side of the ER the signal peptide is cleaved off by the signal peptidase and the protein is folded by the help of luminal chaperones. *Post-translational (right part)*. Proteins with N-termini that are not recognized by the SRP in spite of a functional signal peptide are translated to completion in the cytosol, but their folding is prevented by the interaction with cytosolic Hsp70 proteins. The preprotein interacts with the Sec61 complex in the ER membrane and becomes translocated across the membrane by ATP driven pulling mechanism exerted by luminal chaperones. Inside the ER the preproteins are processed by a signal peptidase (SP) and the proteins fold with the help of chaperones. Protein complexes are indicated in capital letters, proteins are indicated according to the nomenclature used in this manuscript.

In spite of major differences between the import mechanisms of the above-mentioned organelles, the key steps are similar. Receptor proteins select suitable cargo proteins by specific interaction with targeting signals, but this selection can occur either during translation or after translation and can act either on unfolded or folded proteins (Table 1). In all cases, the receptor initiates the interaction of the cargo protein with a complex translocation machinery that can involve the receptor protein(s) itself. Moreover, all cargo proteins are translocated through pore-like structures, but this occurs either in an unfolded linear state or as fully folded protein. After transport the N-terminal sequences encoding targeting signals are processed by specific peptidases within the organelles. Each receptor protein mediates the import of many proteins, which necessitates a recycling of these receptor proteins. The targeting signals for mitochondria, chloroplasts, and the ER are encoded within N-terminal sequences with different denominations (*presequence, transit sequence*, and *signal peptide*), whereas *peroxisomal targeting signals* determine proteins for peroxisomes (Table 1). A comparative overview of the import mechanisms for soluble proteins into different organelles is depicted (Figure 1) and highlights the major steps of protein import. For further details of the import mechanism the readers are referred to excellent reviews that have been published elsewhere [peroxisomes (Hettema et al., 2014; Platta et al., 2014) mitochondria (Chacinska et al., 2009; Schulz et al., 2015); chloroplasts (Li and Chiu, 2010), and ER (Akopian et al., 2013; Johnson et al., 2013b)].

**Table 1:** **Transport processes mediated by the N-terminal targeting signal**.

**Organelle**	**Peroxisome**	**Mitochondria**	**Chloroplast**	**ER** **Co-translational**	**ER** **Post-translational**
**Targeting signal**	**PTS2**	**Presequence**	**Transit peptide**	**Signal peptide**	**Signal peptide**
**Structure**	Amphiphilic α-helix	Amphiphilic α-helix	Amphiphilic α-helix	Hydrophobic α-helix	Less hydrophobic α-helix
**Consensus sequence**	Yes	No	No	No	No
**Linker domain**	Yes	No	No	No	No
**Processing of the N-terminus**	Yes	yes	yes	yes	Yes
**Number of cargo proteins^a^**	< 30	> 1000	> 1000	> 1000	Unclear
**Receptor**	Pex7 Soluble	Tom20 Membrane bound	Toc34/159 Membrane bound	SRP-complex Soluble	
**Translocon^b^**	Pex14/Pex5	Tom40	Toc75	Sec61	Sec61
**Ribosomes**	Free	Free	Free	Membrane bound	Free
**Transfer to organellar membrane**	Co-receptor mediated	Chaperone mediated	Chaperone mediated	SRP-mediated	Chaperone mediated
**Import**	Post-translational	Post-translational	Post-translational	Co-translational	Post-translational
**Protein state** (During translocation)	Folded	Unfolded	Unfolded	Unfolded	Unfolded
**Import mode**	Globular	Linear (N → C)	Linear (N → C)	Linear (N → C)	Linear (N → C)
**Energy^c^**					
Protein translocation	ATP	ATP	ATP	GTP	ATP
Transfer of the targeting signal		Δψ	GTP		
Energy consuming process	Receptor recycling	Protein translocation	Protein translocation	Protein translocation	Protein translocation
**Processing peptidase^d^**	PPP/GPP	MPP	SPP	SP	SP

*Protein transport mediated by N-terminal targeting signals*.

a *Protein numbers are a rough estimation for complex animal or plant organisms based on the assumption that more than at least half of the organellar proteins are soluble: mitochondria (Homo sapiens, Pagliarini et al., 2008), chloroplasts (Arabidopsis thaliana, Richly and Leister, 2004), ER and secretory apparatus not considering the secreted proteins (Rattus norvegicus, Gilchrist et al., 2006) and peroxisomes (Arabidopsis thaliana, Reumann et al., 2007), but in the latter only a third of the proteins encodes a PTS2*.

b *Translocon: proteins forming the pore forming unit for the translocation of the preproteins*.

c *Energy is consumedin form of ATP hydrolysis (ATP), GTP hydrolysis (GTP), or derived from the electrochemical gradient (Δψ)*.

d *Processing peptidases: PPP/GPP, peroxisomal, or glyoxysomal processing peptidase; MPP, mitochondrial processing peptidase; SPP, stromal processing peptidase; SP, signal peptidase*.

### Protein import into peroxisomes

All soluble peroxisomal proteins are encoded by nuclear DNA, produced by free ribosomes and folded in the cytosol before they are translocated across the membrane (Figure 1A) (Léon et al., 2006). This folding might include co-factor binding and oligomerization. Even cross-linked proteins and labeled gold particles up to a size of 9 nm can be imported (Walton et al., 1995; Subramani, 2002; Léon et al., 2006). Peroxisomal proteins harbor a peroxisomal targeting signal (PTS), which is encoded by a peptide sequence either at the extreme C-terminus (type 1, PTS1) (Gould et al., 1988) or proximal to the N-terminus (type 2, PTS2) (Swinkels et al., 1991; Osumi et al., 1991), although, sporadically proteins have been described in peroxisomes that do not encode any of these sequences. The import of a protein requires the interaction of the PTS1 with the soluble receptor protein Pex5^2^ (peroxin 5, Distel et al., 1996; Van der Leij et al., 1993; Dodt et al., 1995; Wiemer et al., 1995; Kragler et al., 1998) or of the PTS2 with the soluble receptor protein Pex7 (Marzioch et al., 1994; Braverman et al., 1997; Woodward and Bartel, 2005). Cargo-loaded receptor proteins translocate to the peroxisomal surface and interact with the docking complex (DC), which is part of the peroxisomal import machinery (PIM) (Figure 1A). The primary docking of Pex5 is driven by a lipid-protein interaction (Kerssen et al., 2006), but the functional interaction is dependent on specific sequences within Pex5 that mediate the interaction with proteins of the DC (Saidowsky et al., 2001; Otera et al., 2002). In contrast, Pex7 cannot move to the peroxisomal surface by itself, but requires the interaction with a co-receptor protein, which encodes the sequence elements required for the interaction with the proteins of the docking complex (Schliebs and Kunau, 2006; Grunau et al., 2009; Kunze et al., 2015). This co-receptor function for Pex7 is exerted in many organisms (metazoa and plants) by the PTS1 receptor Pex5 (Braverman et al., 1998; Otera et al., 1998; Khan and Zolman, 2010), whereas in fungi independent proteins exist for this function (Purdue et al., 1998; Titorenko et al., 1998). Cargo binding was found to be a prerequisite for the interaction of human Pex7 with its co-receptor protein Pex5 (Mukai and Fujiki, 2006; Kunze et al., 2015) and, thus, only cargo-loaded Pex7 can be transported to peroxisomes (Kunze et al., 2015). This resembles the cargo-induced translocation of Pex5 in PTS1-mediated import (Gouveia et al., 2003b).

At the docking complex, both import pathways converge, and thus, will be discussed together highlighting only specific differences. Cargo bound Pex5 integrates into the peroxisomal membrane in an ATP-independent step that is probably driven by protein-protein interactions (Oliveira et al., 2003). During this process Pex5 interacts with the N-terminus of Pex14 proteins via several copies of a conserved sequence motif involving two aromatic amino acids (Schliebs et al., 1999), which fits to the overrepresentation of Pex14 in the membrane complex involving Pex5 (Gouveia et al., 2000). Interestingly, not only the C-terminal part of Pex5 that encodes the cargo-binding domain reaches into the peroxisomal matrix (Gouveia et al., 2003a), but also the N-terminal sequence (Dammai and Subramani, 2001). Similarly, Pex7 is imported into the peroxisomal matrix in an ATP-independent step that causes a complete enclosure of the receptor within the organelle (Rodrigues et al., 2014). Pex5 accumulates at the peroxisomal surface, integrates into the membrane and becomes part of a multiprotein complex before it is recycled (Dodt and Gould, 1996). The mechanism of cargo protein translocation across the peroxisomal membrane has not been resolved, but probably involves a dynamic pore-like core-structure consisting of Pex5 and Pex14 proteins. In reconstitution experiments utilizing protein complexes isolated from peroxisomal membranes and embedded into artificial lipid membranes, the addition of cargo bound receptor protein Pex5 is accompanied by a transient increase in ionic membrane permeability supporting the existence of a pore-like structure (Meinecke et al., 2010). The mechanisms by which receptor proteins release their cargo into the peroxisomal lumen are still unclear, but while in yeast the release of PTS1-carrying cargo from Pex5p involves of the yeast specific peroxin Pex8 (Ma et al., 2013), in mammals the release of cargo proteins is stimulated by a fragment of Pex14 (Freitas et al., 2011). After Pex5 has released its cargo, the receptor protein is recycled back to the cytosol to be available for another round of matrix protein import and possibly to create space for further integration of cargo loaded Pex5. This involves ubiquitination of a conserved cysteine close to the N-terminus of Pex5, the ATP-dependent extraction of Pex5 from the protein complex within the peroxisomal membrane and, finally, the removal of the ubiquitin moiety to regenerate a soluble and cargo-free receptor Pex5 (Platta et al., 2005; Francisco et al., 2014). Ubiquitination is exerted by specific machinery involving an ubiquitin conjugating enzyme (UBC, type E2) and a ubiquitin ligase (type E3). In yeast the UBC activity is exerted by Pex4 (Wiebel and Kunau, 1992; Platta et al., 2007) attached to the outer side of the peroxisomal membrane and the ubiquitin ligase activity is performed by the peroxisomal membrane proteins Pex10 and Pex12 (Platta et al., 2009, 2014). In mammals three homologous cytosolic proteins (UbcH5a-c) exert the UBC activity (Grou et al., 2008), whereas the ubiquitin ligase activity requires the peroxisomal proteins Pex2, Pex10, and Pex12, which might cooperatively exert the ligase activity (Francisco et al., 2014). The extraction of the mono-ubiquitinated Pex5 is exerted by the receptor extraction module (REM) consisting of a peroxisomal transmembrane protein (Pex26/Pex15) and two members of the AAA-ATPase family (Pex1 and Pex6), which utilize the energy of ATP hydrolysis to extract Pex5 from the membrane (Costa-Rodrigues et al., 2004; Platta et al., 2005) (Figure 1A) (for review see, Francisco et al., 2014; Platta et al., 2014). Deubiquitination of Pex5 is exerted by deubiquitinating enzymes (Usp9x/Ubp15) (Debelyy et al., 2011; Grou et al., 2012). Importantly, membrane binding and integration of Pex5 neither requires ATP hydrolysis nor the cysteine in Pex5 nor a functional extraction module. This suggests that the whole energy demand of the peroxisomal import cycle is consumed during receptor extraction. Pex7 necessitates its interaction with the co-receptor not only for its import into peroxisomes, but also for its recycling that depends on the extraction of the co-receptor (Hensel et al., 2011; Liu and Subramani, 2013; Rodrigues et al., 2014). In most organisms, PTS2 carrying proteins are processed inside peroxisomes by the peroxisomal processing peptidase (PPP) releasing a prepeptide harboring the PTS2 (Helm et al., 2007; Kurochkin et al., 2007; Schuhmann et al., 2008). This peptidase is not only required for a functional processing, but also for a continuous peroxisomal protein import (Mizuno et al., 2013). Cytosolic chaperones are involved in the folding of cargo proteins before their transport, but should not be required inside peroxisomes.

### Protein import into mitochondria

More than 99% of mitochondrial proteins are encoded by nuclear genes and translated by free ribosomes. Most soluble mitochondrial matrix proteins contain a targeting signal within their N-terminal amino acid sequence, termed the *presequence*, which mediates the interaction with membrane-bound receptor proteins that are part of a multi-protein complex, the *translocon of the outer mitochondrial membrane* complex (TOM complex; Tom20, 22, 40, 70, 5, 6, 7) (Figure 1B) (Table 1) (Neupert and Herrmann, 2007). Although proteins destined for the mitochondrial matrix are transported after translation is completed (post-translational), their folding is prevented by cytosolic chaperones of the Hsp70 family, because mitochondrial proteins are imported in an unfolded state. These chaperones and other proteinaceous factors of the 14-3-3 family (MSF, mitochondrial import stimulatory factor) support the translocation of preproteins to the mitochondrial membrane (Deshaies et al., 1988; Murakami et al., 1988; Hachiya et al., 1993; Komiya et al., 1997). There, the preprotein interacts sequentially with the mitochondrial receptor proteins Tom20 and Tom22 via different elements of its presequence (Brix et al., 1997; Saitoh et al., 2007). These receptors mediate the transfer of the presequence to the pore forming protein Tom40 (translocon) that channels the preprotein across the outer mitochondrial membrane in a linear mode from the N- to the C-terminus (N→ C) (Model et al., 2008). This transfer is probably driven by the increasing affinity of the presequence to different components of the TOM-complex (*acid chain hypothesis*), which also involves a domain of Tom22 in the intermembrane space (Komiya et al., 1998; Kanamori et al., 1999). In the intermembrane space, the presequence interacts with the *translocon of the inner mitochondrial membrane* complex (TIM complex; Tim23, 50, 17, 21). First, the presequence binds to the primary receptor protein Tim50 (Yamamoto et al., 2002; Mokranjac et al., 2009) and is then transferred to the channel forming protein Tim23 (Truscott et al., 2001) that also interacts with the preprotein (Alder et al., 2008). During protein translocation TOM and TIM complexes are transiently linked to facilitate the transfer of a polypeptide across the double membrane (Chacinska et al., 2005; Tamura et al., 2009). The translocation of the presequence across the inner membrane is driven by the electrochemical force across this membrane (ΔΨ) acting on the positive charges of the presequence (Schleyer et al., 1982; Martin et al., 1991). The subsequent translocation of the complete polypeptide is facilitated by a dragging mechanism from the luminal side of the inner mitochondrial membrane enforcing the directionality of the import process. When the preprotein appears at the inner side of the Tim23 pore it is grasped by intramitochondrial Hsp70 proteins (mtHsp70). This requires the interaction of Tim23 with a multi protein complex, the ATP-coupled import motor (PAM, presequence translocase associated motor: Tim44, 14, 16, mtHsp70, Mge1), on the inner side of the membrane (Neupert and Herrmann, 2007). Within this protein complex the mtHsp70 proteins interacts with the preprotein in an ATP dependent manner, which prohibits sliding back of the preprotein (Neupert and Brunner, 2002). Further import of the preprotein exposes additional sequences that are again covered by mtHsp70 causing a net-onward movement either by ATP hydrolysis or simply due to the avoidance of backsliding (Neupert and Brunner, 2002). Inside the mitochondrial matrix, the preprotein is processed by the mitochondrial processing peptidase (MPP) releasing the N-terminal sequence (Gakh et al., 2002; Teixeira and Glaser, 2013). Finally, the processed protein is folded inside mitochondria by specific chaperones of the Hsp60 family (Cheng et al., 1989; Ostermann et al., 1989).

### Protein import into chloroplasts

Chloroplast proteins that are encoded in the nucleus are equipped with a targeting signal within the N-terminal amino acid sequence termed *transit peptide* (Bruce, 2000). The proteins are synthesized in the cytosol and remain in an unfolded state until they interact with membrane bound receptor proteins at the surface of chloroplasts (Figure 1C) (Schleiff and Becker, 2011). This is supported by cytosolic chaperones of the Hsp70 protein family (Flores-Pérez and Jarvis, 2013) that cooperate either with proteins of the Hsp90 family that bind non-phosphorylated transit peptides and dock at a specific protein of the outer chloroplasts membrane (Toc64) (Qbadou et al., 2006; Fellerer et al., 2011) or with proteins of the 14-3-3 family that specifically bind to phosphorylated sequences within the transit peptides (Waegemann and Soll, 1996; May and Soll, 2000) (for review see, Lee et al., 2013). At the chloroplast surface, the transit peptide interacts sequentially with the receptor proteins Toc34 and Toc159 (Ma et al., 1996; Sveshnikova et al., 2000; Smith et al., 2004), which are only representatives of larger receptor protein families (Toc33 and Toc90, 120, or 132) (Jelic et al., 2003; Smith et al., 2004). All members can contribute to protein import, but have been characterized with different profiles of transit peptide recognition (Kubis et al., 2004; Demarsy et al., 2014). These receptor proteins are part of a large protein complex (TOC: translocon of the outer envelope of chloroplasts, TOC34, 159, 75, 64, 12) involving the specific binding protein for Hsp90 proteins (Qbadou et al., 2006) and the pore-forming Toc75 protein that performs the translocation of the transit peptide across the chloroplast outer membrane (Hinnah et al., 2002). Importantly, both chloroplast receptor types are GTPases that can form homo- and heterodimers via their GTPase domain and they are able to couple nucleotide hydrolysis with the binding of transit peptides and a change in the dimerization status (Smith et al., 2002; Sun et al., 2002; Rahim et al., 2009). Accordingly, non-hydrolyzable GTP interferes with protein import (Schnell et al., 1994; Young et al., 1999), although the GTPase activity of individual receptor proteins is dispensable (Agne et al., 2009; Aronsson et al., 2010). Preproteins are handed over to the translocon Toc75 by a well-defined cycle of events, in which both receptor proteins change their dimerization status, their interaction partner and the phosphorylation state of the bound guanine nucleotide. The transit peptide opens the Toc34 dimer, stimulates its GTPase activity, and initiates its heterodimerization with Toc159, which is prerequisite for the transfer of the transit peptide to Toc159 (Paila et al., 2015). The transit peptide has to be dephosphorylated to bind to Toc159 and the sequence recognized by Toc159 overlaps with the Toc34 binding site, although it is not identical (Schleiff et al., 2002; Becker et al., 2004; Smith et al., 2004; Lee et al., 2009). Finally, the transit peptide is transferred by Toc159 in its GTP-bound state to the translocon Toc75 (Wang et al., 2008), which opens for the translocation of the transit peptide upon GTP hydrolysis in Toc159 (Schleiff et al., 2003). The transit peptide directly interacts with Toc75 (Perry and Keegstra, 1994; Hinnah et al., 2002), but then reaches through the Toc75 channel to interact with a chaperone in the intermembrane space (IAP70, Schnell et al., 1994; Ma et al., 1996), which supports the transfer of the preprotein across the outer envelope membrane. Next, the transit peptide interacts with Tic22 (Kouranov et al., 1998) and finally with a protein complex in the inner membrane (TIC: translocon of the inner envelope membrane; Tic100, Tic214, Tic56, Tic20/Tic21, and Tic40) that mediates the translocation of the preprotein across the chloroplast inner envelope membrane (Kikuchi et al., 2013; Nakai, 2015; Paila et al., 2015). This protein complex can appear with slightly different components, but shares Tic20 (Kouranov et al., 1998; Kovács-Bogdán et al., 2011) or its functional homolog Tic21 (Teng et al., 2006), and Tic110 (Heins et al., 2002). These proteins have been suggested as the key components of the TIC channel and have been directly linked to the channel function (Heins et al., 2002; Balsera et al., 2009). The stromal part of Tic110 interacts with transit peptides as they emerge from the pore (Inaba et al., 2003). Moreover, it forms a platform together with the membrane-bound co-chaperone Tic40, which links the pore with a complex protein machinery that supports preprotein import. This machinery consists of Hsp90 (Inoue et al., 2013), the motor chaperone Hsp93 (Chou et al., 2003, 2006), and the stromal Hsp70 (CpHsp70) (Latijnhouwers et al., 2010). The latter two proteins interact directly with transit peptides *in vitro* (Ivey et al., 2000) and a lack of these proteins interferes with preprotein import (Su and Li, 2008, 2010). Protein import into chloroplasts requires GTP hydrolysis during the early steps of transit peptide insertion (Young et al., 1999), but the translocation of the whole preprotein is driven by ATP hydrolysis by stromal chaperones and partially of a chaperone in the intermembrane space (Flügge and Hinz, 1986). When soluble proteins reach the stroma they are processed by the stromal processing peptidase (SPP) (Richter and Lamppa, 1998; Trösch and Jarvis, 2011) and protein folding is supported by members of the Hsp60 family (Cnp60, chaperonin 60) (Lubben et al., 1989; Kessler and Blobel, 1996).

### Protein import into the ER

Soluble proteins that are determined for an insertion into the ER harbor an N-terminal signal peptide, which is often cleaved off upon import (Blobel and Dobberstein, 1975; Schatz and Dobberstein, 1996). However, the recognition of the signal peptide can occur either during translation inducing a translational arrest until the ribosome has docked to the ER (cotranslational protein import) or after translation is completed (post-translational protein import) requiring the contribution of cytosolic chaperones that retain the proteins in an import competent unfolded state (Figure 1D) (Walter and Lingappa, 1986; Zimmermann et al., 2011; Johnson et al., 2013b). The choice of the transport route is influenced in the yeast by the hydrophobicity of the targeting signal (Ng et al., 1996) and in metazoa by the size of the protein (Johnson et al., 2013a).

The *co-translational protein import* is initiated by the interaction between the signal peptide and the soluble signal recognition particle (SRP) representing the cognate receptor protein. This SRP is a GTP-hydrolyzing ribonucleoprotein complex comprised of one (prokaryotes) or more (e.g., six in metazoa) proteinaceous components and an RNA (Akopian et al., 2013). This system is functionally equivalent to the bacterial protein export machinery and many contributions have been initiated by findings in this field. One domain (M-domain) of the key subunit (Srp54) exerts the binding to the signal peptide (Clemons et al., 1999), whereas the other domain (NG-domain) mediates the interaction with the membrane-bound docking site (SRP receptor, SR) (Schwartz and Blobel, 2003; Halic et al., 2004). The SRP and the SR contain GTPase domains and interact via these domains (Akopian et al., 2013). The recognition of a signal peptide occurs within a large protein complex consisting of the ribosome, the nascent chain of the cargo protein appearing at the ribosomal exit tunnel [together forming the ribosome nascent chain complex (RNC)] and the SRP scanning the N-terminus of the newly synthesized protein. A suitable signal peptide initiates a conformational change in the SRP that stalls translation and allows the interaction of the SRP with the membrane bound SR at the ER surface (docking site). In eukaryotes this SR is a heterodimer consisting of a soluble α- and a membrane bound β-subunit (Tajima et al., 1986; Schwartz and Blobel, 2003), which is directly linked to the Sec61 complex involving the pore forming Sec61α protein (translocon) (Wiedmann et al., 1987). Both the SRP and the SR contain GTPase modules that mediate their interaction, but also regulate the interaction between these protein complexes by switching between the GTP- and GDP-bound state (Focia et al., 2004). GTP-bound SR binds to cargo-loaded SRP and hydrolysis of SR bound GTP is coupled to the release of the SRP into the cytosol for recycling. Moreover, the rate of GTP hydrolysis in the SRP affects the interaction time with the RNC, the attachment of the SRP-RNC complex at the ER membrane and the release of the signal peptide from the SRP. In addition, structural rearrangements within this large protein complex occur independently of local conformational changes upon GTP hydrolysis, which generate a complex cycle of events. During this process the RNC is transferred from the SRP to the Sec61 complex, which initiates the insertion of the nascent chain into the Sec61α channel and the sealing of Sec61α by the ribosome. Finally, GTP hydrolysis by the SRP is associated with a conformational change that initiates the release of the SRP from the ribosome, which allows the resumption of translation for an efficient coupling of protein synthesis and the transport of the newly synthesized protein across the ER membrane.

*Post-translational protein import* acts on proteins that pass the scan of the N-terminal sequence by the SRP, e.g., because hydrophobicity is below a certain threshold. These proteins remain unfolded and translocate independently to the surface of the ER, which requires cytosolic proteins of the Hsp70 and the Hsp40 family (Chirico et al., 1988; Dierks et al., 1993; Ngosuwan et al., 2003). There, the signal peptide interacts with the Sec61 translocon (Johnson et al., 2012) and releases cytosolic chaperones (Plath and Rapoport, 2000). In yeast, the transfer across the membrane is exerted by the Sec61 complex (αβγ) in cooperation with additional proteinaceous factors that have been described as Sec62/Sec63 complex (Panzner et al., 1995), which is comprised of Sec62p, Sec63p, Sec71p, and Sec72p (Lyman and Schekman, 1997). However, the latter two proteins are not essential and absent in mammals. In contrast to co-translational protein import, the energy for translocation is provided by luminal chaperones of the Hsp70 family (Kar2p/Grp78/BiP) that bind to the Sec62/63 complex and pull preproteins through the Sec61 channel, which renders the process ATP-dependent (Hansen et al., 1986).

Thus, the energy required for preprotein translocation following the initial transfer of the signal peptide is provided either by the GTPase activity of the ribosome during translation, which pushes the linear protein through the Sec61-translocon (co-translational) or by the ATPase activity of the luminal chaperone (Kar2p/Grp78/BiP) that drags the proteins into the ER. Two models have been suggested to account for the directionality of the translocation, which is accomplished by luminal chaperones. Either the chaperone utilizes the energy of ATP hydrolysis to exert a series of individual dragging steps or it progressively covers those parts of the preprotein, which appear at the luminal side and thereby prohibits the back-slipping of the preprotein (Elston, 2002). Independently of the import mode, the N-terminal signal peptide is cleaved off by a peptidase (signal peptidase) (Weihofen et al., 2002) in the ER lumen and a variety of luminal chaperones assist the folding of the protein within the ER (Braakman and Bulleid, 2011).

### Comparison between the transport routes

Altogether, the mechanisms of protein transport from the cytosol into peroxisomes, mitochondria, chloroplasts, and the ER differ remarkably, but the import can be initiated by targeting signals proximal to the N-terminus (PTS2 for peroxisomes), which become processed during or after the import. Peroxisomal protein import differs from other import mechanisms in several important aspects: (i) It acts on fully folded proteins, whereas the post-translational import routes and the import into the ER all translocate proteins in an unfolded state; (ii) Cytosolic chaperones are required for protein folding, but are not as essential for protein transfer to the peroxisomal membrane as they are for post-translational import into mitochondria, chloroplasts or the ER; (iii) The peroxisomal receptor proteins (Pex5 and Pex7) are predominantly soluble like the SRP, whereas the other receptor proteins (Tom20 and Toc34) are membrane bound and receive the majority of proteins via chaperone assisted transfer. (iv) The peroxisomal import of folded proteins needs a flexible pore with large diameter, which is provided by the dynamic cooperation of Pex14 with the receptor Pex5, whereas the translocon structures of mitochondria (Tom40), chloroplasts (Toc34), or the ER (Sec61α) have a small, but defined diameter and permit the channeling of unfolded linear proteins across the membrane; (v) Cargo-loaded peroxisomal receptor proteins (Pex5 and Pex7) integrate into the membrane and reach into the organellar lumen to release their cargo proteins inside peroxisomes, which requires an energy-consuming extraction of the receptor to recycle it to the cytosol; (vi) ATP hydrolysis for receptor extraction is the sole nucleotide triphosphate-consuming step of peroxisomal import, whereas the transfer of unfolded proteins through the translocons of mitochondria, chloroplasts, and the ER requires intraorganellar ATP hydrolysis by chaperones to pull the preproteins into the organelle. In addition, the forward motion of translation that is driven by GTP hydrolysis provides energy for the co-translational import into the ER. These processes are distinct from the energy consumption for the pathfinding of N-terminal signals, which involves GTP hydrolysis for the signal peptides (ER) and transit peptides (chloroplasts). (vii) As peroxisomal proteins are imported in a folded state, they do not require extensive folding inside the organelle, whereas all other organelles have an elaborate folding machinery inside.

## Targeting signals and their receptors

Targeting signals have been described as amino acid sequences necessary and sufficient for the proper localization of a protein, which emphasizes the functional properties of these sequence elements. Alternatively, targeting signals could be defined by their ability to mediate an interaction between the protein harboring the signal and a receptor protein, which is required to initiate protein transport across a specific organellar membrane. The majority of soluble proteins enclosed in mitochondria, chloroplasts or the ER harbor targeting signals that are all encoded within the N-terminal region of the protein. Within the target organelle, a short N-terminal fragment including the targeting signal is cleaved off the protein. The type 2 peroxisomal targeting signal (PTS2) was known to resemble these targeting signals with regard to its position within the primary sequence of the protein and to the intraperoxisomal processing. However, recent investigations elucidated the structural properties of the PTS2 and its binding mode to its receptor, which revealed further similarities to other N-terminal targeting signals. In contrast, the PTS1 resides at the extreme C-terminus and is recognized by another receptor protein. Thus, we mainly compare the properties of the N-terminal targeting signals and only briefly touch on PTS1, because the comparison of the two peroxisomal targeting signals will be required in later chapters.

### Peroxisomal targeting signals

Although the two *peroxisomal targeting signals* (PTS1 and PTS2) have been amply described in various organisms and their receptor proteins have been identified, individual soluble peroxisomal proteins have been identified that do not encode any of these signals. This has originally been attributed to a potential third type of peroxisomal targeting signals (PTS3), but this signal has never been characterized and the import either depends on a PTS-independent interaction with a receptor protein (Klein et al., 2002) or on co-import of proteins (piggy-back), which is a specific property of peroxisomal import (Yang et al., 2001; Subramani, 2002; van der Klei and Veenhuis, 2006; Islinger et al., 2009).

#### PTS2 and its interaction with the receptor protein Pex7

**Figure 2:**
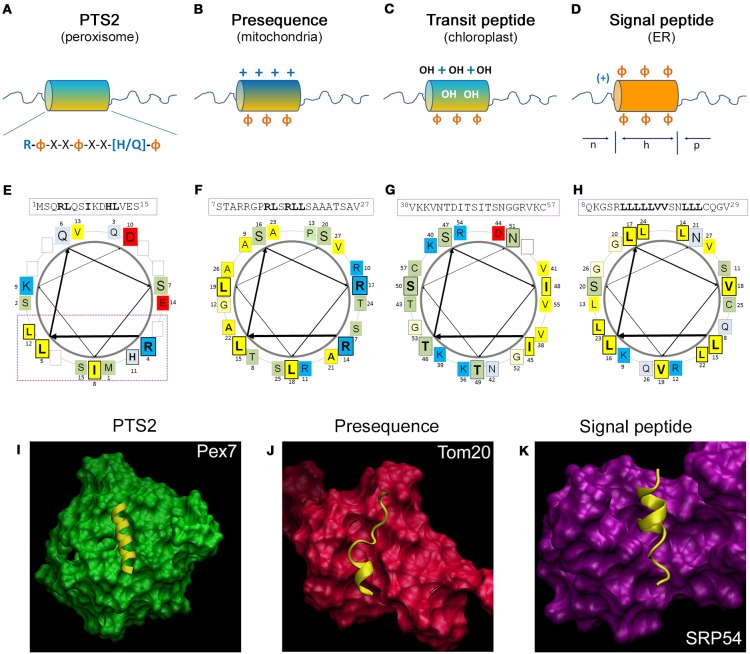
**Comparison of the structural properties of N-terminal targeting signals and their interaction with the receptor proteins. (A–D)** *Schematic representation of the N-terminal amino acid sequences encoding different targeting signals*: **(A)** the peroxisomal PTS2 forming an α-helical domain encoding the consensus sequence, which is followed by an unstructured sequence element; **(B)** the mitochondrial presequence is enriched for positive charges and forms an amphipathic α-helical domain, **(C)** the chloroplast transit peptide sequence is enriched in hydroxylated amino acids; and **(D)** the signal peptide for the ER is composed of a positively charged (n)-domain, a hydrophobic (h)-domain, and a polar (p)-domain. **+,** positive charges; OH, hydroxylated residues; Φ, hydrophobic residues; *orange*, hydrophobic side; *blue*, hydrophilic side of the helix. **(E–H)** *Helical wheel depiction of typical N-terminal targeting signals*: **(E)** the PTS2 of yeast thiolase (*Sc*Fox3), **(F)** the presequence of rat aldehyde dehydrogenase (*Rn*Aldh2), **(G)** the transit peptide of pea ribulose-1,5-bisphosphate carboxylase/oxygenase small subunit (*Ps*prSSU), and **(H)** the signal peptide of bovine preprolactin (*Bt*PRL). The amino acid sequences depicted in the α-helical wheel projections are indicated above using the numbering of the primary sequence; amino acids of the central turn are indicated by larger letters; residues of the PTS2 consensus sequence, residues of the presequence interacting with Tom20, the hydroxylated residues of the transit peptide and the hydrophobic patch of the signal sequence are indicated bold and boxed. The color code for the physical properties of the residues is as follows: *acidic* red, *basic* blue, *hydrophobic* yellow, *polar basic* bluish gray and *polar neutral* green. The arrows indicate the progression of the amino acid sequence within the α-helical wheel. **(I–K)** *3D structure of the receptor protein and the* α*-helix of the targeting signal:* **(I)** the N-terminus of yeast Fox3 involving a PTS2 (yellow) together with the receptor protein Pex7 (green), **(J)** the presequence of rat Aldh2 (yellow) together with the soluble domain of Tom20 (red), **(K)** the leader peptide of yeast dipeptidylpeptidase B (yellow) together with the cargo binding domain of archeal Srp54. The structures have been generated by the program *visual molecular dynamics* (VMD) (Humphrey et al., 1996) based on the datasets PDB:3W15 (Pan et al., 2013) (I), PDB:1OM2 (Abe et al., 2000) (J) and PDB:3KL4 (Janda et al., 2010) **(K)**.

The observation that a peroxisomal targeting signal is encoded in proximity to the N-terminus of the rat peroxisomal enzyme thiolase led to the identification of the PTS2 (Osumi et al., 1991; Swinkels et al., 1991), which was later also identified in yeast and plants (Gietl et al., 1994; Glover et al., 1994). The consensus sequence has originally been described as (R/K)-(L/V/I)-*X*_5_-(Q/H)-(L/A)^4^ (Figure 2A) highlighting two conserved dipeptide motifs separated by five arbitrary amino acids, which are sensitive to different point mutations (Glover et al., 1994; Tsukamoto et al., 1994). Later on, this motif was extended to R-(L/V/I/Q)-*X-X-*(L/V/I/H)-(L/S/G/A)-*X-*(H/Q)-(L/A) based on a compilation of the most common PTS2 variants (Petriv et al., 2004). This suggested a previously unrecognized conservation at the central amino acid *X*_3_, which was consistently found to present with large and hydrophobic properties (Petriv et al., 2002; Reumann, 2004; Kunze et al., 2011). In a reporter construct harboring the N-terminus of rat thiolase, the functionality of the PTS2 was destroyed by a substitution of residue *X*_3_ with a negatively or positively charged amino acid (Kunze et al., 2011). Based on the sequence of charged/polar and hydrophobic residues, an α-helical structure with two turns was suggested, which orients all key residues of the consensus sequence toward one side of this helix (Figure 2E). Moreover, PTS2 motifs are highly enriched in amino acids overrepresented in helical structures and the introduction of the helix-breaking amino acid proline at the least conserved position of a prototypical PTS2 abrogated its functionality (Kunze et al., 2011). This was in line with previous suggestions of a helical structure for PTS2 motifs based on the paucity of proline residues within PTS2 motifs (Reumann, 2004) and the observation that a PTS2-destroying point mutation in the rat thiolase N-terminus generated a mitochondrial targeting signal *de novo* (Osumi et al., 1992). Finally, this suggestion was confirmed by the elucidation of the 3D structure of the N-terminus of the yeast ortholog of thiolase (Fox3) in a receptor bound state, in which the PTS2 non-apeptide presented as α-helix (Pan et al., 2013). Altogether, the linear PTS2 non-apeptide corresponds to an α-helix, in which one flank is occupied by the key residues that align amino acids of the same property. When comparing the N-terminal sequences of PTS2-containing proteins, the region upstream of the PTS2 was found enriched in acidic residues (Reumann, 2004; Kunze et al., 2011), whereas the region downstream of the PTS2 contains many amino acids, which are typical for unstructured stretches (Kunze et al., 2011). The latter probably reflects a linker domain, which serves the exposure of the PTS2 helix from the fully folded core protein. Accordingly, a similar linker domain has been described next to the PTS1 (Neuberger et al., 2003c), but was not observed in proteins that are imported in an unfolded state into other organelles. In addition, the flexible linker domain of PTS2-carrying proteins could also be necessary for the exposition of the processing site toward the peptidase inside peroxisomes.

The PTS2 receptor Pex7 has been identified in various organisms as a protein essential for the import of PTS2-encoding proteins (Marzioch et al., 1994; Braverman et al., 1997; Woodward and Bartel, 2005). It belongs to the family of WD40 domain proteins sharing a cone-like shape (Stirnimann et al., 2010). Thus, the structure of the human PEX7 protein has been predicted several times by independent groups (Braverman et al., 2002; Stanley and Wilmanns, 2006; Kunze et al., 2011), although early predictions were hampered by the lack of closely related template structures. However, the identification of the PTS2 binding site within these structures was difficult, until the pattern of evolutionary highly conserved surface residues was taken into account to identify the most important areas of the protein. This allowed the identification of a groove on top of the cone structure of human PEX7, which is covered with residues that are suitable for an interaction with the conserved side of a PTS2 helix (Figure 2I) (Kunze et al., 2011). This prediction was verified experimentally (Kunze et al., 2011) and the elucidation of the 3D structure of yeast Pex7 together with the N-terminus of thiolase confirmed the suggested model (Pan et al., 2013). Thus, the α-helix is located horizontally in a shallow groove on the top side of the Pex7 cone burying about half of the helix. The interaction obtains energetic contributions from several hydrophobic, but also from ionic and hydrogen bonds, which is conserved across evolution from yeast to man. However, this interaction appears to be weak until co-receptor binding transforms the cargo-bound receptor into a stable trimeric complex (Mukai and Fujiki, 2006; Pan et al., 2013; Kunze et al., 2015). This has been originally attributed to a conformational change in the receptor (Mukai and Fujiki, 2006), but the structural resolution of the yeast trimeric complex consisting of Pex7, Fox3, and a part of the yeast co-receptor Pex21 indicated that the co-receptor directly interacts with residues of the PTS2 helix (Figure 2I) (Pan et al., 2013). Furthermore, the interaction of Pex7 and the co-receptor is dependent on the presence of a cargo protein (Grunau et al., 2009; Kunze et al., 2015) and on the ability of Pex7 to bind the cargo protein (Kunze et al., 2015). This suggests that the co-receptor is able to discriminate PTS2-like motifs, which are bound to Pex7, but expose residues with different physical properties from the Pex7 averted side of the helix. Accordingly, Pex7 and its co-receptor could be considered as bipartite receptor, in which Pex7 exerts a preselection of putative cargo proteins, which are subsequently approved by the binding of the co-receptor. Such mechanism would enlarge the area of the receptor that scans a PTS2 motif and increases the number of residues encoding targeting information.

#### PTS1 and its interaction with the receptor protein Pex5

The PTS1 has been identified as peroxisomal targeting signal mediating the import of firefly luciferase into peroxisomes of monkey cells (Gould et al., 1987). The signal is located at the extreme C-terminus of the protein (Gould et al., 1988) and the minimal targeting signal has been narrowed down to a tripeptide consisting of serine, lysine, and leucine (–SKL) or conserved variants thereof (Gould et al., 1989; Swinkels et al., 1992). Later on, it was found that the interaction with the receptor protein Pex5 is also severely affected by the preceding sequence (Lametschwandtner et al., 1998) that mediates flexibility for a proper exposure of the PTS1 from the folded core protein (Neuberger et al., 2003a; Brocard and Hartig, 2006).

Proteins encoding a PTS1 interact with the receptor protein Pex5 (Van der Leij et al., 1993; Dodt et al., 1995; Wiemer et al., 1995; Kragler et al., 1998) via a tetratricopeptide repeat (TPR) domain covering the C-terminal half of the receptor (Brocard et al., 1994; Dodt et al., 1995). The structure of the TPR resembles a bent half-pipe (Gatto et al., 2000), into which the last three amino acids of the cargo proteins insert and thereby induce a conformational change (Stanley et al., 2006; Fodor et al., 2015).

### Mitochondrial targeting signals

Research on the N-terminal part of mitochondrial preprotein sequences (*presequence*) encoding the mitochondrial targeting signal revealed that these sequences do not present with a conservation pattern at the level of the primary amino acid sequence, which could be converted into a consensus sequence. However, these sequences share preferences in physicochemical properties and the frequency of individual amino acids such as an overrepresentation of positively charged residues and, more specifically, of arginine, whereas negatively charged residues are nearly absent (Figure 2B) (von Heijne et al., 1989; Huang et al., 2009). Accordingly, mitochondrial targeting signals can be generated quite easily *de novo* by mutations (Vassarotti et al., 1987) or insertion of arbitrary amino acid sequences at the N-terminus of a protein (Lemire et al., 1989). Moreover, these sequences contain elements with a high propensity to form α-helices with amphipathic properties, in which hydrophobic residues cover one side and positively charged residues the other side of the helix (Roise et al., 1986; von Heijne, 1986). The α-helical element of the rat aldehyde dehydrogenase (Aldh2) presequence, which binds the cytosolic part of the receptor Tom20, consists of a six amino acid core element (^14^RLSRLL^19^) (Abe et al., 2000; Muto et al., 2001) (Figure 2F). Comparison of mitochondrial presequences revealed the conserved pattern φ*χχφφ*, in which φ represents a bulky hydrophobic residue and χ indicates any amino acid (Obita et al., 2003), although substitutions of φ by alanine are partially tolerated (Mukhopadhyay et al., 2006).

The elucidation of the 3D structure of cargo-loaded Tom20 (Abe et al., 2000; Saitoh et al., 2007) revealed that the amphipathic helix of the presequence lays within a broad, shallow binding groove consisting of a four helix bundle (Figure 2J). The hydrophobic residues comprising one side of the amphipathic helix reach into the hydrophobic binding groove of Tom20, whereas the positive charges of the presequence interact with negatively charged residues at the border of the binding groove (Abe et al., 2000). Thus, the interaction between signal and receptor is mediated by hydrophobic and ionic interactions, although it appears insensitive to the salt concentration (Brix et al., 1997). Interestingly, a peptide can interact with Tom20 in more than one binding state, which fits with a certain degree of mobility of the peptide within the binding groove and the acceptance of divergent peptides as interaction partners (Saitoh et al., 2007, 2011).

### Chloroplast targeting signals

The N-terminal sequences of soluble chloroplast proteins, called *transit peptides* (Bruce, 2000), encode targeting information, which involves binding motifs for receptor proteins of the Toc34 and the Toc159 family and binding sites for Hsp70 (Rial et al., 2000; Zhang and Glaser, 2002) and Hsp90 proteins (Qbadou et al., 2006). Moreover, specific sites within transit peptides facilitate their phosphorylation, which has not been observed in mitochondrial presequences (Waegemann and Soll, 1996; May and Soll, 2000), but is required for the interaction with 14-3-3 proteins (May and Soll, 2000). Transit peptides show a characteristic amino acid distribution, but a consensus sequence cannot be delineated from primary sequences of naturally occurring transit peptides (Bruce, 2001). This is in line with a high promiscuity of the import system for arbitrary N-terminal peptides. Naturally occurring transit peptides are rich in hydroxylated amino acids (von Heijne et al., 1989), whereas negative charges are underrepresented and, in contrast mitochondrial presequences, arginines are not overrepresented (von Heijne et al., 1989) (Figure 2C). On a helical wheel prediction, typical transit peptides encode a domain, which shows amphipathic properties due to a hydrophobic and a positively charged hydrophilic patch on opposite sides of the α-helix, but between these elements polar wedges of hydroxylated residues and occasionally negativly charged residues seem to be present (Bruce, 2000) (Figure 2G). The structure of the transit peptide of ribulose bisphosphate carboxylase (Rubico) activase from the green algae *Chlamydomonas reinhardii* has been resolved confirming the α-helical domain (Krimm et al., 1999). However, transit peptides are predominantly unstructured in aqueous environment (Bruce, 1998; Krimm et al., 1999), which fits to their amino acid distribution (von Heijne and Nishikawa, 1991), but in hydrophobic environment the fraction of α-helical elements increases (Endo et al., 1992; Bruce, 1998; Krimm et al., 1999). However, these common properties of all transit peptides are complemented by more specific ones, which allow the discrimination of transit peptides by different members of the Toc159 receptor family (Jelic et al., 2003; Demarsy et al., 2014; Dutta et al., 2014). This is compatible with the observation that within a transit peptide the binding sites for Toc34 and Toc159 are only partially overlapping leaving space for receptor discrimination. Thus, the relative affinity of a transit peptide to different receptor proteins determines the transport route of the encoding protein into different types of plastids.

At the chloroplast surface, transit peptides interact with the receptor Toc34 in a first step and, subsequently, with different members of the Toc159 receptor family. The first resolution of the 3D structure of pea Toc34 identified the GTP binding domain within the overall structure of the receptor (Sun et al., 2002), whereas more recent investigation studied the monomeric and dimeric state of the receptor (Koenig et al., 2008). In the latter study, a groove was identified in proximity to the GTP binding site, which has been proposed as transit peptide binding site (Koenig et al., 2008). However, a 3D structure of Toc34 together with a transit peptide is not available and, thus, cannot be presented here (Figure 2).

### Targeting signals for the ER

The signal determining a protein for the import into the ER/secretory apparatus has been already described in 1981 (Kreil, 1981). Detailed analysis of available signal sequence revealed that signal peptides are usually rich in hydrophobic residues with a core element composed of a positively charged domain, a hydrophobic domain of 8–12 amino acids and a polar C-terminal region, which have been denominated as [n]-domain, [h]-domain, and [c]-domain (Briggs and Gierasch, 1984; von Heijne, 1985; Gierasch, 1989) (Figure 2D). Individual changes in the charge pattern of the [n]-domain or of the [c]-domain had little effect, whereas a shortening of the [h]-domain had severe consequences for the import of a reporter protein (Nilsson et al., 2015) and the presence of several positive charges in the [c]-domain was also detrimental (Fujita et al., 2011). According to their hydrophobic character signal peptides are often not soluble in water, but form α-helical domains in hydrophobic environment (Briggs and Gierasch, 1984; Yamamoto et al., 1990), which can be depicted on a helical wheel projection for a typical signal peptide (Figure 2H). The importance of the hydrophobic helical element is further supported by detrimental effects of a single charged and helix breaking residue within the [h]-domain (Bruch et al., 1989; McKnight et al., 1989; Rothe and Lehle, 1998). However, in contrast to previous assumptions (Bird et al., 1987), the hydrophobic properties alone are not directly correlated with the quality of the signal peptides, and an excess of hydrophobic residues was found detrimental for signal peptides (Huber et al., 2005). A comparison of naturally occurring signal peptides could not delineate a conservation pattern that allows the definition of a consensus sequence. Accordingly, the signal sequences are often resistant to mutations (Gierasch, 1989) and many arbitrarily generated N-terminal sequences can act as signal peptides (Kaiser et al., 1987) similar to the signals recognized by the chloroplast and mitochondrial import systems.

The 3D structure of the ligand-binding domain of the SRP has been first resolved for Srp54 of *Thermus aquaticus* (Keenan et al., 1998), but later on also the M-domain of the human Srp54 protein has been resolved (Clemons et al., 1999). Moreover, these complexes were analyzed together with the nascent chain bound ribosome and the SRP receptor (Halic et al., 2004, 2006). However, the interaction between the SRP and a signal peptide has only been elucidated with high resolution for archaeal SRPs (Janda et al., 2010; Hainzl et al., 2011). The binding site for the signal peptide is composed of four helices that form a groove, which is limited on one side by the finger domain of the RNA. The binding groove is covered with hydrophobic residues with mobile side chains, especially methionines, supporting the flexibility in cargo selection (Bernstein et al., 1989). Moreover, more than one binding mode for signal peptides have been obtained in archeal Srp54 proteins (Janda et al., 2010; Hainzl et al., 2011). We depict the archaeal Srp54 structure together with a signal peptide (Figure 2K) (Janda et al., 2010) in spite of the evolutionary distance between archaea and eukaryotes, because the structural conservation between the protein complexes (RNC-SRP-SR) has recently been demonstrated (Halic et al., 2006) and the eubacterial Srp54 homolog can even be functionally integrated into the mammalian SRP (Bernstein et al., 1993). Illustratively, this depiction demonstrates the similarity of the binding mode of a signal peptide to Srp54 proteins with that of other targeting signals their receptor proteins.

However, co-translational protein import is only one path into the ER, whereas post-translational import is independent of the recognition of a signal peptide by the SRP. Thus, the existence of two alternative pathways suggests that certain properties of the signal peptides specify them for one of these transport routes, although all N-terminal amino acid sequences that successfully mediate the import of the encoding protein into the ER are considered signal peptides. The co-translational transport route requires the early recognition of the signal peptide upon its appearance at the ribosomal exit site, whereas the post-translational transport route skips this recognition, but the protein needs to remain unfolded. Accordingly, in yeast the hydrophobicity of the signal peptides was suggested as primary determinant favoring co-translational protein import (Ng et al., 1996), whereas in multicellular animals the post-translational protein import appears restricted to small proteins (Johnson et al., 2012, 2013a).

### Comparative summary

The PTS2 and targeting signals for soluble proteins of mitochondria, chloroplast or the ER share their position within an N-terminal sequence element that is cleaved upon import into the target organelle and the involvement of an α-helical domain that mediates the interaction with the receptor protein. However, the targeting signals for mitochondria, chloroplasts, and the ER are highly diverse and relatively robust against single amino acid substitutions. Moreover, these signals can be easily generated *de novo*, whereas the PTS2 has a clear consensus sequence consisting of five key positions which are sensitive to amino acid substitutions. The composition of the complete N-terminal sequences shows characteristic patterns for each organelle, but in case of the PTS2 the unstructured domain following the consensus sequence appears most obvious. The α-helical elements of the signals bind to the receptor proteins in a similar mode with one side of the helix embedded into a binding groove on the receptor surface (Figures 2I–K). However, in Pex7 the binding groove is narrower compared to the other receptors, which is in agreement with its binding of peptides with a well-defined consensus sequence, whereas Tom20 and Srp54 require more flexibility to enable binding of peptides with variable primary sequence. Moreover, the helical element encoding the PTS2 (I) appears longer when compared to that encoding the presequence (J) or the signal peptide (K), although on average the α-helical elements should have comparable length (Gierasch, 1989; Moberg et al., 2004; Kunze et al., 2011; Nilsson et al., 2015). However, this might be due to the tight cargo binding of Pex7 in the presence of the co-receptor (not shown), which forces the peptide into a well-defined structure. In contrast, only a short sequence element of the presequence or of the signal peptide has to be in a helical conformation, whereas the larger binding groove of Tom20 or Srp54 might be compatible with other forms of cargo binding. In all cases, the major fraction of the interaction area between α-helix and receptor protein is covered by hydrophobic residues, whereas ionic interactions are restricted to the edges of the binding groove. A characteristic of the PTS2-Pex7 interaction is the contribution of the co-receptor protein that enlarges the interaction area and increases the affinity.

## Similarity of targeting signals and the specificity of protein transport

**Figure 3:**
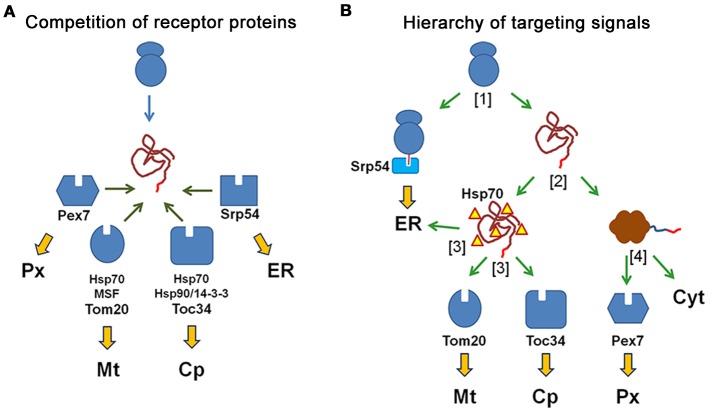
**N-terminal targeting signals determine the transport route of proteins by the interaction with the receptor proteins**. **(A)** *Competition of receptor proteins*: the N-terminal amino acid sequence of a newly synthesized protein can interact with all receptor proteins, which compete for the peptide sequence (peroxisomal Pex7, mitochondrial Tom20, chloroplast Toc34, and Srp54 for the ER) and with additional cytosolic proteins that might affect these interactions (Hsp70, Hsp90, 14-3-3 proteins). The choice of the transport route is based on the relative affinity of the peptide sequence to different receptor proteins. **(B)** *Different import mechanisms generate a hierarchy of targeting signals*: An N-terminal amino acid sequence is sequentially scanned by diverse receptor proteins, because these interactions occur at different time points during the production and folding of the protein. A newly synthesized protein either binds to the SRP to become translated into ER or it finishes translation in the cytosol [1] Next, the protein either becomes folded or remains unfolded due to its interaction with chaperones, [2] Unfolded proteins can interact with the mitochondrial receptor Tom20, the chloroplast receptor Toc34 or the Sec61 complex of the ER (translocon), [3] Finally, folded proteins can either interact with the soluble receptor protein Pex7, which initiates their transport into peroxisomes, or they remain in the cytosol [4].

Although the N-terminal targeting signals for mitochondria, chloroplasts, the ER and also for peroxisomes (PTS2) are structurally similar, the accurate distribution of proteins between different subcellular compartments demonstrates that protein transport is highly specific. At first glance, the N-terminal amino acid sequence of a newly synthesized protein is concomitantly exposed to all available receptor proteins, which compete for the N-terminal sequence (Figure 3A). Accordingly, the specificity of protein transport can only be achieved by promoting the interaction between an N-terminal amino acid sequence and its appropriate receptor protein, whereas interactions with undesired receptor proteins that would induce mistargeting must be avoided. However, in reality the different receptor proteins scan an N-terminal amino acid sequence during distinct phases of protein formation, because of the different mechanisms of protein import (Figure 3B). The recognition of a signal can occur either directly upon its appearance at the exit site of the ribosome (signal peptide), or after translation, when the unfolded protein reaches the organellar membrane (presequence, transit peptide, signal peptide) or after completion of protein folding (peroxisomal targeting signal). This implicates that an early decision in favor of one transport route might exclude other routes that are initiated by receptor interactions at a later stage of protein formation. Thus, the properties of the protein import machineries modulate the specificity of protein transport, although the relative affinity of an N-terminal amino acid sequence to different receptor proteins remains a crucial determinant for the choice of the transport route.

### Relative affinity of targeting signals to different receptor proteins

The effectivity of the interaction between an amino acid sequence and a receptor protein should correlate with the quality of this sequence as targeting signal. This gets even more important under conditions, when different receptor proteins compete for the same amino acid sequence and, thus, the relative affinity of this sequence for diverse receptor proteins appears as key determinant for targeting specificity. In this case, the fitting between a targeting signal and the signal binding domain of its cognate receptor protein should be much better than with any other receptor protein, which favors the formation of the desired receptor-cargo interaction (*positive discrimination*). However, the idea of tight fitting is inconsistent with the conspicuous degeneration of targeting signals and the high portion of hydrophobic residues in the interaction domains, which render a specific interaction less plausible. Moreover, the basic interaction strength between a mitochondrial presequence and Tom20 (Abe et al., 2000) or between the N-terminus of a PTS2-carrying cargo protein and PEX7 is surprisingly weak (Mukai and Fujiki, 2006; Pan et al., 2013; Kunze et al., 2015), which is in good agreement with the low number of amino acids involved in this interaction. In contrast, the interaction strength between phosphorylated transit peptides and the chloroplast receptor Toc34 is drastically higher (Sveshnikova et al., 2000).

Alternatively, the specificity for a single binding partner could originate from the existence of individual residues within targeting signals that exclude an interaction with competing receptor proteins by their physico-chemical properties (*negative discrimination*). Such a mechanism could account for the specificity of PTS2 motifs, because individual point mutations in a prototypical PTS2, which retain peroxisomal targeting, allow concomitant alternative targeting (Kunze et al., 2011). Moreover, the interaction of an amino acid sequence with a receptor could also be modulated by sequences or residues in proximity to the direct binding site, which might exert additional stabilizing or repulsive effects. Altogether, the necessary difference in the affinity to different receptor proteins can originate either from specifically strengthening the desired interaction or from disfavoring the interaction with other receptor proteins. However, the discriminatory power is most probably the product of a co-evolution of targeting signals and available receptor proteins, which is supported by the observation that a plant chloroplast protein is targeted to mitochondria, when ectopically expressed in yeast cells (Hurt et al., 1986).

Focusing on the short amino acid segments directly interacting with the receptor proteins might cause a disregard of the surrounding amino acid sequences that are also part of the processed N-terminal sequence. As these sequences are cleaved off, they do not contribute to protein function and should be flexible for adaptation processes. Moreover, these sequences encode information for the binding of chaperones in mitochondrial presequences and chloroplast transit peptides (Zhang and Glaser, 2002) or for phosphorylation sites within transit peptides that mediate the interaction with 14-3-3 proteins (Waegemann and Soll, 1996; May and Soll, 2000). Therefore, it is remarkable that the sequence preceding the PTS2 motif was found enriched in negative charges (Reumann, 2004), whereas mitochondrial presequences are rich in positive charges and depleted of negative ones (Pujol et al., 2007). The similarity of the targeting signals for mitochondria and for chloroplasts have been long recognized starting with glutathione reductase from pea (Creissen et al., 1995) and has been amply investigated since then. A direct comparison of presequences and transit peptides revealed an overrepresentation of positive charges in presequences and of hydroxylated residues in transit peptides (Jarvis and Robinson, 2004). This study was extended by a combination of bioinformatic and mutational approaches (Pujol et al., 2007) and even a restraint to the residues at the extreme N-terminus of the proteins showed characteristic differences (Bhushan et al., 2006). This suggests that general properties of the whole N-terminal sequence (presequence, transit peptide or signal peptide) influence the quality of a targeting signal specified by the domain directly interacting with the receptor protein. These properties are probably shaped by evolutionary processes and can be used by prediction algorithms that successfully discriminate N-terminal targeting signals (Emanuelsson et al., 2007; Mitschke et al., 2009).

### Coupling of independent recognition steps

Although the direct interaction between the targeting signal and the receptor protein is a key step in the initiation of protein import, the implementation of an additional recognition event, which secondarily scans already chosen targeting signals, can provide a selectivity filter function to improve specificity. Such additional evaluation of a targeting signal is compatible with the formation of a trimeric complex consisting of targeting signal, receptor, and a third protein as well as with a hand-over mechanism, in which the cargo protein is further processed by a second protein. However, both mechanisms benefit from the involvement of additional sequence motifs within or in proximity to the targeting signal, which do not participate in the primary binding of the receptor protein. Accordingly, sequence elements that are not directly involved in receptor binding should be able to modulate the import efficiency of a protein. Exemplarily, the co-receptor protein for the PTS2 receptor Pex7 drastically stabilizes the interaction between this receptor and its cargo (Mukai and Fujiki, 2006; Pan et al., 2013; Kunze et al., 2015) and the 3D structure of the yeast trimeric complex (Pex7, Pex21, N-terminus of Fox3) indicates a direct interaction between residues of the co-receptor and of the PTS2 (Pan et al., 2013). This contribution of the co-receptor increases the area of the PTS2 helix, which is available for the recognition of a PTS2 by the receptor/co-receptor complex. However, the sequential assembly of the trimeric complex (Kunze et al., 2015) suggests that the co-receptor interacts with a preformed PEX7-cargo dimer and, thus, the binding of the co-receptor acts as independent quality control of the preformed dimeric complex. At the mitochondrial membrane, the presequence not only interacts with Tom20, but also with the second receptor protein Tom22 (Brix et al., 1997). However, the presequence binds Tom20 predominantly via hydrophobic interactions, whereas the interaction with Tom22 is mainly dependent on ionic interactions (for discussion see, Endo and Kohda, 2002). On the chloroplast surface, members of the Toc159 family bind to a sequence element of the transit peptide, which only partially overlaps with the Toc34 binding site, and thereby independently evaluate transit peptides after their primary recognition by Toc34. Moreover, phosphorylation is a frequently observed property of transit peptides that increases their affinity for the chloroplast receptor Toc34 (Sveshnikova et al., 2000), although the lack of phosphorylation sites did not change the specificity of targeting (Nakrieko et al., 2004). However, this phosphorylation also allows the interaction with proteins of the 14-3-3 family, which support the transport of the preprotein to the chloroplast surface together with Hsp70 proteins (May and Soll, 2000). Finally, the binding of a signal peptide to the bacterial homolog of SRP induces a conformational change within this protein, but the quality of the signal peptide markedly correlates with the velocity, at which the first intermediate state is reached (Zhang et al., 2010). Furthermore, a good signal peptide delays GTP hydrolysis by the GTPase activity of the SRP, which extends the time window during which the trimeric complex of nascent chain, ribosome, and SRP can reach the ER membrane (Zhang et al., 2010). Altogether, the different import routes all involve mechanisms that add such selectivity filters.

### Differences in the import mechanism pose a hierarchy of targeting signals

**Figure 4:**
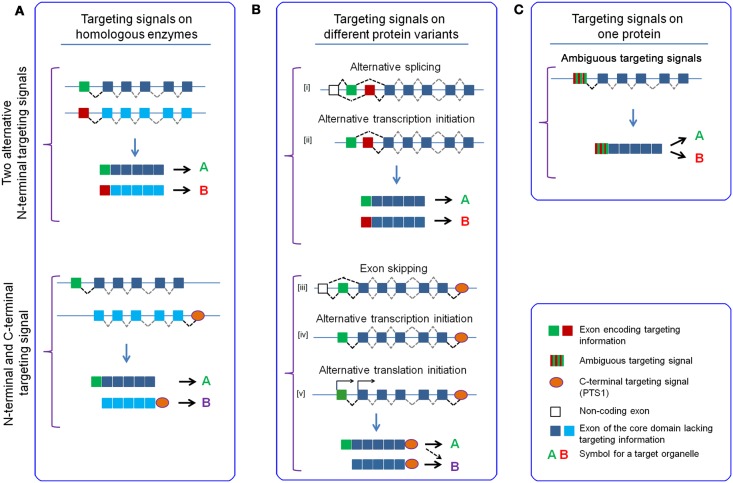
**Mechanisms to provide the same enzymatic activity or protein function within different subcellular compartments**. Bilocalization of protein(s) requires the presence of two alternative targeting signals, which can be either encoded by alternative N-terminal sequences (*upper part*) or can be encoded by an N-terminal targeting signal and a C-terminal PTS1, respectively (*lower part*). **(A)** Two independent genes code for proteins with the same enzymatic activity of function (isoenzymes/homologoues), which harbor different targeting signals. **(B)** Two variants of the same protein are generated from a single gene, which share the core domain(s), but differ in the encoded targeting signals; variants encoding alternative N-terminal sequences can be obtained by [i] alternative splicing from the same pre-mRNA with a non-coding first exon or [ii] by alternative transcription initiation generating alternative first exons, which use to the same splice acceptor site of the second exon. Variants with and without N-terminal targeting signal, but sharing a C-terminal PTS1 can be generated by [iii] exon skipping behind the first non-coding exon, which omits the second exon encoding the N-terminal targeting signal, [iv] alternative transcription initiation ablating the first exon and [v] alternative translation initiation at different start codons within the same mRNA. **(C)** One protein is equipped with an ambiguous targeting signal, which is sufficient to mediate the concomitant targeting of the protein to more than one organelle.

In contrast to the mechanisms listed above, which improve the fidelity of transport route selection by the choice of the appropriate receptor protein(s), the transport routes themselves are also ranked by the distinct phases of protein formation, during which a particular receptor scans the N-terminal amino acid sequence of a newly generated protein. This is equally important for the choice of the transport route, because it renders the alternative import mechanisms unequal (Figure 3B). The entrance into the ER is triggered by signal peptides directly after the appearance of the nascent chain at the exit site of the ribosome. These peptides are recognized by the SRP, which acts as soluble receptor. However, this interaction also induces translational stalling, which prevents the synthesis of the residual protein until the SRP-bound signal sequence has been transferred to the translocon (Sec61 complex). Thus, the newly translated protein is directly guided into the ER, whereas other targeting signals that might be also encoded within the protein sequence are never accessible in the cytosol. This renders co-translational import into the ER dominant over all other transport routes (Figure 3B; [1]). The translation of other proteins, which have not been sequestered by the SRP, is completed in the cytosol generating folding competent polypeptides. However, only a fraction of these polypeptides is actually folded, whereas proteins encoding a mitochondrial presequence, a chloroplast transit peptide or a signal peptide for post-translational ER import remain in an unfolded state due to binding of various chaperones of the Hsp70 family (Figure 3B; [2]). These unfolded proteins can interact with membrane-bound receptors on the surface of mitochondria, chloroplasts, or the ER and the transfer to the organellar membrane can be accelerated by cytosolic factors such as mitochondrial import stimulatory factor (MSF) (Hachiya et al., 1993) or 14-3-3 proteins for chloroplasts (May and Soll, 2000). Properties that distinguish mitochondrial and chloroplast preproteins have been elucidated (Huang et al., 2009), but the import mechanisms do not suggest a hierarchical relation between these targeting signals (Figure 3B; [3]). In contrast, peroxisomal, nuclear, and cytosolic proteins are folded in the cytoplasm with the help of folding chaperones. However, proteins exposing a peroxisomal targeting signal either at their N-terminus (PTS2) or its C-terminus (PTS2) bind to cytosolic receptor proteins and become imported into peroxisomes (Figure 3B; [4]).

Altogether, in this concept the choice of an import route is the consequence of temporarily distinct decisions, in which the different receptor proteins interfere with one step in the production of a folded protein. Accordingly, an early route decision can exclude a protein from all transport pathways that are chosen at a later stage, which implements a hierarchy of transport routes reflected by the hierarchy of targeting signals (Neuberger et al., 2004). This idea is supported by the analysis of naturally occurring proteins encoding a functional PTS1, which revealed that various proteins located exclusively in mitochondria or the ER sometimes encode a functional PTS1 that is not utilized (Neuberger et al., 2004). This suggests that in these cases, an evolutionary selection preventing undesired peroxisomal targeting is not required, whereas cytosolic proteins are sensitive to the addition of PTS1 motifs. Moreover, *de novo* generated mitochondrial targeting signals can suppress naturally occurring PTS1. This is exemplified in the human enzyme alanine:glyoxylate aminotransferase (AGXT) involved in peroxisomal glyoxylate detoxification. A mutation generating a mitochondrial tartgeting signal causes the mistargeting of an otherwise intact enzyme from peroxisomes to mitochondria, which is sufficient to cause a clinical picture of primary hyperoxaluria type 1 (OMIM #259900) similar to the loss of enzymatic activity (Danpure, 2006). This highlights the clinical importance of the hierarchical ranking of targeting signals. Moreover, the importance of the folding state for the choice of the import route was investigated by the use of a reporter protein (dihydrofolate reductase, DHFR), which can be forced into a folded state by a pharmaceutical compound (methotrexate). When this reporter protein was equipped with a mitochondrial targeting signal and a PTS1, it is exclusively found in mitochondria. However, when protein folding was favored by the addition of methotrexate, this led to peroxisomal targeting of the reporter protein corroborating the concept of a hierarchy of targeting signals (Mukhopadhyay et al., 2004). It remains to be clarified, whether the dominance of the mitochondrial targeting signal is solely caused by an efficient avoidance of protein folding or whether the late exposure of the PTS1 during translation also contributes to the subordination of the PTS1. In the latter case, the N-terminal position of the PTS2 might offer a possibility to (partially) overcome the hierarchy of targeting signals.

Importantly, this hierarchy of targeting signals implicates that the specificity of protein import is primarily dependent on the ability to make certain crucial decisions during protein formation, which are only partially determined by the relative affinity of different receptor proteins to the same amino acid sequence.

### Additional levels of regulation

In addition to the mechanisms that support a high specificity of protein transport at the level of cargo recognition, further cell biological processes might support this specificity. One promising candidate is the enrichment of mRNA encoding organellar proteins in proximity to these organelles. Such mRNA enrichment has been described for fractions containing predominantly peroxisomes (Zipor et al., 2009), mitochondria (Kaltimbacher et al., 2006; Eliyahu et al., 2010), chloroplasts (Weis et al., 2013), or the ER (Reid and Nicchitta, 2015), but only the latter was independent of translation (Pyhtila et al., 2008; Jagannathan et al., 2014).

In summary, several mechanisms supposedly act in concert to facilitate the specificity of transport processes in spite of the similarity of N-terminal targeting signals.

## Dual targeting and bilocalization of proteins

As specific targeting signals initiate the transport of proteins to distinct subcellular compartments, a tight relation between the primary sequence and the subcellular location of proteins was supposed, which resulted in the assumption of a predefined distribution of all proteins. Any deviation from a discrete location such as the occurrence of minor protein fractions in other compartments was attributed either to contaminations indicating the imperfectness of the isolation procedure (e.g., density gradient centrifugation) or to insufficiencies of the detection tool (e.g., low of antibody specificity). This assumption was corroborated by the observation that the concurrent presence of the same enzymatic activity in different subcellular compartments is often achieved by the existence of homologous proteins (isoenzymes), which encode different targeting signals.

However, more recently, the number of reports describing real *bilocalization* of individual proteins by *dual targeting* has been steadily increasing, which has been summarized for proteins localized in peroxisomes and other organelles (Ast et al., 2013), mitochondria and chloroplasts (Small et al., 1998; Carrie and Small, 2013), or secretory proteins and other organelles (Porter et al., 2015). These observations were sometimes made by accident, but more often were facilitated by modern techniques such as the detailed analysis of subcellular fractionation by advanced mass spectrometric methods (e.g., protein correlation profiling), which allows a better discrimination of organellar constituents from contaminants (Andersen and Mann, 2006; Foster et al., 2006; Wiese et al., 2007), or by the systematic investigation of EGFP-fusion proteins (Li et al., 2006; Carrie et al., 2009). An obvious biological advantage of such bilocalization of a single protein is genomic efficiency, because the number of genes that are required to supply different organelles with the same protein function is reduced. This is most obvious when considering the dual targeting of about 100 proteins to mitochondria and chloroplasts, many of which are involved in organellar DNA replication and protein synthesis (Carrie and Small, 2013). However, the savings due to bilocalization of proteins probably require a complex arrangement of targeting information, because the presence of two targeting signals alone might not be sufficient for dual targeting. Many targeting signals are positioned within the N-terminal part of the encoding proteins, which share organelle specific properties. Whereas, these differences support specificity of targeting signals by interfering with competing transport routes, they might pose a problem for the performance of dual targeting. Moreover, the hierarchy of targeting signals can also prevent dual targeting of proteins that encode two targeting signals, because even targeting signals that are positioned at different ends of a protein can negatively affect each other, such as the dominance of N-terminal targeting signals over the C-terminal PTS1.

The concurrent presence of a protein function or protein activity within different subcellular compartments can be achieved by various means (Figure 4). In the traditional concept, the bilocalization of a protein function is realized by *independently encoded homologous proteins* that are equipped with different targeting signals (Figure 4A). These signals can either be both located at the N-termini of the proteins (*upper part*) or at opposite ends (*lower part*). Alternatively, the cell can produce different protein variants (isoforms) derived from one gene that share the core domain, but differ slightly in their primary sequence, which is sufficient to exchange targeting signals (Figure 4B). In this process, either variants with alternative N-terminal amino acid sequences are generated that differ by the encoded targeting signal (*upper part*) or variants are produced that share a C-terminal PTS1, but encode or lack an additional N-terminal targeting signal (*lower part*). Protein variants with alternative N-terminal sequences (*upper panel*) can be generated from a single gene by the production of different mRNAs that are obtained either by alternative splicing of the same pre-mRNA or by alternative transcription initiation based on different promoters that generate different pre-mRNAs (Mueller et al., 2004; Yogev and Pines, 2011). Protein variants that encode targeting signals at the opposite ends of the protein probably necessitate the omission of the N-terminal targeting signal to disclose a functional PTS1 (*lower panel*). Thus, the two protein variants should differ in the absence or presence of the N-terminal targeting signal, which can be achieved by the omission of the N-terminal part of the protein sequence either by alternative translation initiation or leaky ribosome scanning (Elgersma et al., 1995; Wamboldt et al., 2009), next to the abovementioned mechanisms of alternative splicing and alternative transcription initiation (Ast et al., 2013).

Finally, an increasing number of reports describe *dual targeting* of a protein, which means the transport of the identical protein into different subcellular compartments. These proteins harbor an *ambiguous targeting signal* (Small et al., 1998; Silva-Filho, 2003; Yogev and Pines, 2011) that induces the concomitant transport to alternative destinations by overlapping targeting signals (Figure 4C). Such targeting signals have been predominantly found in plant proteins bilocalized to mitochondria and chloroplasts (Carrie and Small, 2013; Baudisch et al., 2014) and use the traditional import pathways into these organelles (Langner et al., 2014). The amino acid composition of N-terminal sequences encoding ambiguous targeting signals show properties of both targeting signals, which emphasizes the intermediate state of such peptides (Pujol et al., 2007). However, it should be mentioned that protein transport into mitochondria and chloroplasts is especially suitable for such a mechanism, because the import route into these organelles is highly similar involving chaperones that keep the proteins in an unfolded state within the cytosol before the proteins bind to membrane bound receptors.

## Two independent peroxisomal targeting signals as evolutionary advantage

The import machinery of peroxisomes for soluble proteins can accept two completely independent types of targeting signal due to two receptor proteins with specific cargo binding domains, although the transport routes converge at an early stage of the import process. This could be an evolutionary heritage tracing back to ancient developments of eukaryotic cells, but, surprisingly, some organisms lack the whole PTS2 mediated import pathway (Motley et al., 2000; Gonzalez et al., 2011; Faust et al., 2012). However, the possibility to encode targeting signals at different termini of a protein could also pose an advantage under specific conditions. Especially, those properties of one targeting signal, which allow the performance of an irreplaceable function might account for the increased fitness of organisms that have two targeting signals at their disposal. In this context, the position of the PTS2 next to the N-terminus and its structural similarity with other N-terminal targeting signals might confer a functional distinction between the two types of peroxisomal targeting signals.

The appearance of a second targeting signal for peroxisomes could have been relevant during a specific phase of evolution, in which novel N-terminal targeting signals occurred, such as the era after the endosymbiontic uptake of purple bacteria and cyanobacteria as protomitochondria and protoplastid, which later developed to mitochondria and chloroplasts, respectively (Dyall et al., 2004). In this time period, many genes were relocated from the organellar genome to the nucleus, which required the establishment of novel protein import machineries for the endosymbiontic organelles, because the proteins, now encoded by nuclear genes, were produced in the cytosol and had to be imported into mitochondria and chloroplasts. This included the creation of receptor proteins accepting a plethora of targeting signals with variable similarity, which can be easily generated *de novo* and suffice to initiate the translocation of proteins across the organellar membranes. However, these novel transport routes could easily act as competitors for the peroxisomal protein import machinery, particularly when considering that the mechanistic differences render the latter subordinate to the mitochondrial or chloroplast import pathways. This could have caused a detrimental relocation of some peroxisomal proteins comparable to the mislocalization of alanine:glyoxylate aminotransferase (AGXT) in human patients (Section Additional Levels of Regulation) unless the cells were able to reestablish the specificity of protein transport. Under these conditions, the genesis of a second peroxisomal targeting signal could have been a countermeasure in a competitive situation originating from novel import systems utilizing N-terminal targeting signals. Different evolutionary processes are conceivable within such a scenario. The PTS1-mediated import system could have existed before the endosymbiontic events, but might have been overruled and functionally disabled by the dominance of newly generated import systems utilizing N-terminal targeting signals. In such a scenario, the development of an independent peroxisomal targeting signal that is also encoded close to the N-terminus (PTS2) could have been required to perpetuate peroxisomal protein import unless further adaptations enabled the continuation of the original transport route. Alternatively, the PTS2 mediated import pathway could have been the original one, but when this targeting signal was recognized by the receptors of the protein import machineries of mitochondria or chloroplasts, a novel targeting signal close to the C-terminus (PTS1) could have facilitated the abrogation of undesired N-terminal targeting signals without affecting targeting to peroxisomes. Both models suppose the existence of the peroxisomal import system before the appearance of competing import machinery. Alternatively, the co-existence of two independent peroxisomal targeting signals could also present a continuous advantage during evolution. Provided that the similarity between the PTS2 and other N-terminal targeting signals allows the generation of ambiguous targeting signals, which is hardly conceivable for the PTS1, this should allow the bilocalization of the encoding protein. Such ambiguous targeting signals have previously been discussed in the context of dual targeting of proteins to mitochondria and chloroplasts, but could also involve protein transport to peroxisomes and other organelles. This could be an important intermediate step during the change of protein compartmentation, because peroxisomal protein import via the PTS1 is notoriously subordinate to other protein transport routes. Thus, any *de novo* generation of an alternative targeting signal at the N-terminus of a soluble peroxisomal protein encoding a PTS1 should abrogate peroxisomal transport and prohibit bilocalization. Similarly, the *de novo* generation of a PTS1 at the C-terminus of a mitochondrial or chloroplast protein should remain free of consequences, because in this context the novel PTS1 cannot initiate peroxisomal import due to the hierarchy of targeting signals. In contrast, an ambiguous targeting signal that concurrently destines the protein for peroxisomes and another organelle by the same N-terminal amino acid sequence could allow the bilocalization of this protein, which would be an important intermediate step in the exchange of a targeting signals.

## Changes of targeting signals and the subcellular localization in an evolutionary context

In contrast to the presentation in many textbooks, the compartmentation of enzymatic reactions and even of whole metabolic pathways can differ between evolutionary distant organisms. A well-known example is the degradation of the most abundant fatty acids in mitochondria of chordates, which contrasts the exclusively peroxisomal degradation of these fatty acids in yeast and plant species (Poirier et al., 2006; Houten and Wanders, 2010). Less prominent examples are changes in the compartmentation of an individual enzyme, which can occur within relatively short time scales such as the relocation of the glyoxylate-degrading enzyme alanine:glyoxylate aminotransferase (AGXT) (Danpure, 2006). This enzyme has been found exclusively in mitochondria, exclusively in peroxisomes or bilocalized in different mammalian species (Birdsey et al., 2004) and even within the family of bats (*chiroptera*), the localization of the protein differs between species (Liu et al., 2012). The importance of proper targeting of this enzyme for mammalian physiology is highlighted by the inherited human disease hyperoxaluria (type 1), which can originate either from a loss of the enzyme activity (Salido et al., 2012) or from a mistargeting of an otherwise intact enzyme from peroxisomes to mitochondria (Purdue et al., 1990).

Certainly, the presently observable differences in the enzymatic compartmentation between organisms are the product of evolutionary processes, based on which the subcellular distribution of an enzyme has changed over time. This relocation of a protein had to be achieved by an exchange of targeting signals, which is based on stepwise alteration in the primary sequence. Importantly, all intermediate steps of such a development had to be compatible with the functioning of the affected metabolic pathway(s) to fulfill the demands of the organism. Thus, a gradual change of a protein's subcellular location is highly desirable to facilitate concomitant adaptation processes, which is another important application of dual targeting. However, a gradual exchange of targeting signals has to cope with the hierarchy of targeting signals, which might prohibit dual targeting in spite of the presence of two independent targeting signals.

As many targeting signals are encoded close to the N-terminus (PTS2, presequences, transit peptides, and signal peptides), whereas the PTS1 resides at the extreme C-terminus, an exchange of targeting signals either involves two different N-terminal targeting signals or the replacement of an N-terminal targeting signal by a C-terminal one or of a C-terminal targeting signal by an N-terminal one.

The substitution of N-terminal targeting signals can be achieved either by the gradual substitution of single amino acids to convert one targeting signal into another one, or by the replacement of a complete N-terminal sequence module by an amino acid stretch that is encoded by an independent DNA sequence. The latter requires the invention of a novel DNA element encoding an independent amino acid sequence, which has to be integrated into the transcriptional and translational unit of the gene. In the transitional phase the concomitant production of the old and the new protein variant and their transport into different organelles should be important and can be achieved by diverse mechanisms comparable to the examples described above (Figure 4B). In contrast, a process involving the gradual substitution of amino acids offers an ambiguous targeting signal as a suggestive intermediate (Figure 4C). The observation that the N-termini of dually targeted proteins (mitochondria and chloroplasts) unite properties of presequences and transit peptides (Pujol et al., 2007) suggests the feasibility of a gradual change. Whether similar processes are feasible for PTS2 motifs has not been studied yet.

In contrast, the exchange of an N-terminal targeting signal for a PTS1 or *vice versa* within a naturally occurring protein requires independent mechanisms for the generation or inactivation of each of these targeting signals. A *de novo* generation of an N-terminal targeting signal for mitochondria, chloroplasts or the ER can be obtained by an elongation of the protein at its N-terminus using various mechanisms such as the introduction of a start codon in the 5′-UTR or of an alternative transcription initiation site, which all benefit from the high degeneracy of these targeting signals and the efficiency of this process has been described (Kaiser et al., 1987; Vassarotti et al., 1987; Lemire et al., 1989). It should be stressed that such newly generated N-terminal extensions have to encode more than just the minimal receptor binding site, because the N-terminal sequences of naturally occurring preproteins present with additional properties that are characteristic for the organelle or with the ability to interact with cytosolic chaperones. However, most probably these properties need not be perfectly realized in the beginning. The ablation of an existing N-terminal targeting signal can be exerted by the inverse mechanisms such as the inactivation of the first start codon, alternative splicing that skips the exon encoding the start codon together with a part of the N-terminal sequence or the generation of an alternative transcription initiation site.

The position of the PTS1 at the extreme C-terminus renders it suitable for an easy ablation of this signal, but certain properties of this signal facilitate its spontaneous formation as well. The first description of the PTS1 as C-terminal tripeptide in its most prominent form (-SKL) (Gould et al., 1987) revealed the involvement of two amino acids encoded by six different codons (serine and leucine), which renders its *de novo* generation by a statistical event rather probable. Moreover, the apparent degeneracy of the PTS1 (Lametschwandtner et al., 1998; Brocard and Hartig, 2006) further extends the number of arbitrary tripeptides functioning as weak PTS1, which further increases the probability of spontaneous formation. Thus, a novel PTS1 could easily be generated by point mutations within the original protein, but the finding that an unstructured linker domain between the core protein and the PTS1 is important for its functionality (Neuberger et al., 2003a) took this simple model into question. Thus, an alternative mechanism appears more promising, which permits the elongation of the protein by a (partial) read-through of the endogenous stop codon (Freitag et al., 2012; Schueren et al., 2014; Stiebler et al., 2014). This mechanism also generates a novel C-terminal ending and benefits from the relatively high propensity to obtain a PTS1-like sequence by such arbitrary extension. Furthermore, it introduces a short amino acid sequence that can serve as favorable linker domain in front of the PTS1. Conversely, the ablation of a functional PTS1 can easily be accomplished by point mutations or the introduction of a premature stop codon within the linker domain, because this sequence should not contribute to the structure of the core protein. However, the exchange of targeting signals involving a PTS1 is prone to detrimental effects caused by the hierarchy of targeting signals, because the PTS1 is subordinate to N-terminal targeting signals. Accordingly, the *de novo* generation of an N-terminal targeting signal should abrogate the peroxisomal targeting mediated by the original PTS1 and, thus, should prevent bilocalization. Reciprocally, the spontaneous generation of a PTS1 alone is not sufficient to induce peroxisomal targeting of a protein encoding an alternative targeting signal at its N-terminus, which excludes a beneficial effect of the novel PTS1. In this context, the similarity of the PTS2 and other N-terminal targeting signals might represent a functional distinction between the PTS1 and the PTS2, because it is conceivable that PTS2 motifs can be part of an ambiguous targeting signal that concomitantly targets a protein into peroxisomes and another organelle.

## Summary

A specific and efficient transport of proteins from the cytosol into various compartments is a prerequisite for the beneficial effects of sequestering proteins and metabolites into membrane-bound subdomains. The mechanisms of protein import across the confining single or double membrane differ remarkably in the timing of receptor binding, the folding status of the transported protein, the function of the energy consuming steps or the requirement for intraorganellar folding. However, all transport routes are accessible by N-terminal targeting signals that involve an α-helical domain, which interact with the appropriate receptor protein to initiate translocation. In spite of the structural similarity between these N-terminal targeting signals the distribution of the majority of cellular proteins is well-defined, highlighting the specificity of the transport processes. This specificity is enhanced by unique properties of the targeting signals, which render them suitable for a classification into a type of targeting signal (PTS2, presequence, transit peptide, signal peptide), although these targeting signals are not highly conserved, but rather degenerate. These properties are sufficient to discriminate between receptor proteins and thus to select the appropriate transport route. However, the different mechanisms of protein import implicate that the different receptor proteins do not simply compete for the N-terminal sequence of a newly generated protein, but individual receptors can interact solely within a certain time frame during the formation of a fully folded protein. This can be either during translation (ER: co-translational) or after translation, but also before folding starts (mitochondria, chloroplasts, ER: post-translational) or after the folding of the protein (peroxisome). The chronological order of peptide scanning by different receptor proteins is reflected by the hierarchy of targeting signals, because an early decision for one transport route (e.g., mitochondria) excludes the later choice for another organelle (e.g., peroxisomes), which depends on the interaction with another receptor at a later stage. However, the specificity of protein transport does not preclude a bilocalization of proteins by dual targeting, which necessitates the concomitant presence of more than one targeting signal. Such bilocalization increases genetic efficiency, because only one gene can supply protein function within diverse cellular compartments. However, bilocalization can also serve as an important intermediate step during evolutionary adaptation processes involving a redistribution of proteins, because during a transitional phase a continuation of a process at its original location is as important for survival as its invention and optimization at a novel place. In this context, the hierarchy of targeting signals is important, because the presence of two targeting signals is not sufficient if one route is subordinate to the other one.

Interestingly, two functionally equivalent targeting signals can initiate the transport of a soluble protein into peroxisomes (PTS1 and PTS2), which differ by their relation to other targeting signals. The PTS1 is encoded at the extreme C-terminus and appears late during translation, which renders the PTS1 clearly subordinate to the N-terminal targeting signals. In contrast, the PTS2 is structurally similar to other N-terminal targeting signals, which might enable the generation of ambiguous targeting signals. We suggest that this difference might be a crucial advantage for the organism, which favors the coexistence of two peroxisomal targeting signals. The PTS2 is probably more compatible with a bilocalization of the encoding protein, but might be more prone to mislocalization due to its similarity to other targeting signals.

### Conflict of interest statement

The authors declare that the research was conducted in the absence of any commercial or financial relationships that could be construed as a potential conflict of interest.

## Footnotes

^1^As cytosol and nucleus continuously exchange solutes and proteinaceous material, we do not explicate the differences here.

^2^To facilitate reading, we use one nomenclature for proteins from all species using standard abbreviations with the first letter capitalized.

^3^For the sake of clarity some simplifications had to be made concerning the completeness of members of the membrane protein complexes and disregarding the import machineries for membrane proteins.

^4^For the PTS2 consensus sequence we introduced a nomenclature that indicates all residues independently, but highlights the key residues with S and the arbitrary residues with X: S1-S2-X1-X2-X3-X4-X5-S3-S4 (Kunze et al., 2011).

## Abbreviations

PEX: peroxin
RNC: ribosome nascent chain complex
TOM: translocon of the outer mitochondrial membrane
TIM: translocon of the inner mitochondrial membrane
TOC: translocon of the outer chloroplast envelope
TIC: translocon of the inner chloroplast envelope.
